# Naphthoquinone Derivatives with Anti-Inflammatory Activity from Mangrove-Derived Endophytic Fungus *Talaromyces* sp. SK-S009

**DOI:** 10.3390/molecules25030576

**Published:** 2020-01-29

**Authors:** Hongju Liu, Chong Yan, Changqun Li, Tingting You, Zhigang She

**Affiliations:** 1 School of Pharmacy, Guangdong Medical University, 523808 Dongguan, China; jdsbj2000@163.com (C.Y.); lcqstary@163.com (C.L.); youtt2013@163.com (T.Y.); 2 School of Chemistry, Sun Yat-Sen University, 510275 Guangzhou, China

**Keywords:** 1,4-naphthoquinones, *Talaromyces* sp., marine fungus, anti-inflammatory

## Abstract

Twelve 1, 4-naphthoquinone derivatives, including two new (**1** and **2**) and 10 known (**3**–**12**), were obtained from endophytic fungus *Talaromyces* sp. SK-S009 isolated from the fruit of *Kandelia obovata*. All structures were identified through extensive analysis of the nuclear magnetic resonance (NMR), mass spectrometry (MS) and circular dichroism (CD), as well as by comparison with literature data. These compounds significantly inhibited the lipopolysaccharide (LPS)-induced nitric oxide (NO) production in the murine macrophage cell line (RAW 264.7 cells). The half maximal inhibitory concentration (IC_50_) values, except for compound 2, were lower than that of indomethacin (26.3 μM). Compound **9** inhibited the LPS-induced inducible nitric oxide synthase (iNOS) and cyclooxygenase-2 (COX-2) mRNA expressions in RAW 264.7 macrophages. Additionally, compound **9** reduced the mRNA levels of pro-inflammatory factors interleukin (IL)1β, IL-6, and tumor necrosis factor (TNF)-α. The results of this study demonstrated that these 1, 4-naphthoquinone derivatives can inhibit LPS-induced inflammation.

## 1. Introduction

Inflammation is a normal immune process and one of the most important ways to protect the body from infection and tissue injury. However, prolonged or excessive inflammation can lead to a variety of diseases. Many diseases, such as arthritis, inflammatory bowel disease, neurodegenerative disorders, and septic shock syndrome, are related to inflammation. During the inflammatory process, the immune monocytes and macrophages can be stimulated and overexpress pro-inflammatory cytokines, including tumor necrosis factor (TNF)-α, interleukin (IL)-1β, and IL-6, as well as inflammatory factors such as nitric oxide (NO) and prostaglandin E2 (PGE2), produced by inducible nitric oxide synthase (iNOS) and cyclooxygenase-2 (COX-2), respectively [1]. Overproduction of these factors can lead to cell damage and inflammatory disease [2]. Therefore, inhibition of the production of these inflammatory mediators is an effective way to treat the inflammatory diseases [3].

**Figure 1:**
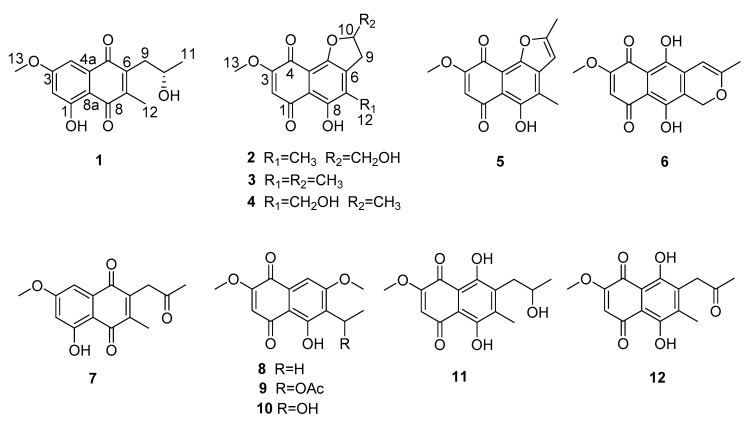
Structures of compounds **1**–**12**.

Marine-derived endophytic fungi are important sources of bioactive compounds [4,5,6,7]. During our investigation of mangrove-derived endophytic fungus as sources of bioactive secondary metabolites, we isolated the endophytic fungus *Talaromyces* sp. SK-S009 from the fruit of *Kandelia obovata*. Organic extracts of rice solid culture showed significant anti-inflammatory activity. Chemical investigation of the extracts led to the isolation of twelve 1, 4-naphthoquinone derivatives (Figure 1). Here, we report the isolation, structure determination, and anti-inflammatory activities of 1, 4-naphthoquinone derivatives (**1**–**12**).

## 2. Results and Discussion

### 2.1. Metabolites Isolation

The EtOAc extract of the fermentation was fractionated by repeated silica gel chromatography and Sephadex LH-20 column chromatography, as well as reversed phase high-performance liquid chromatography (RP-HPLC) to yield twelve 1, 4-naphthoquinone derivatives, including two new (**1** and **2**) and 10 known (**3**–**12**). The structures of the known compounds were identified as anhydrojavanicin (**3**) [8], 2,3-dihydro-5-hydroxy-4-hydroxymethyl-8-methoxy-2-methylnaphtho[1,2-b]furan-6,9-dione (**4**) [9], anhydrojavanicin (**5**) [10], anhydrofusarubin (**6**) [11], 2-acetonyl-3-methyl-5-hydroxy-7-methoxy-naphthazarin (**7**) [12], 6-ethyl-2,7-dimethoxyjuglone (**8**) [13,14], 6-[1-(acetyloxy)ethyl]-5-hydroxy-2,7-dimethoxy-1,4-naphthalenedione (**9**) [15], 5-hydroxy-6-(1-hydroxyethyl)-2,7-dimethoxy-1,4-naphthalenedione (**10**) [16], solaniol (**11**) [17], and javanicin (**12**) [18], by comparison of their spectroscopic data with those reported in the literature.

### 2.2. Structure Identification

**Figure 2:**
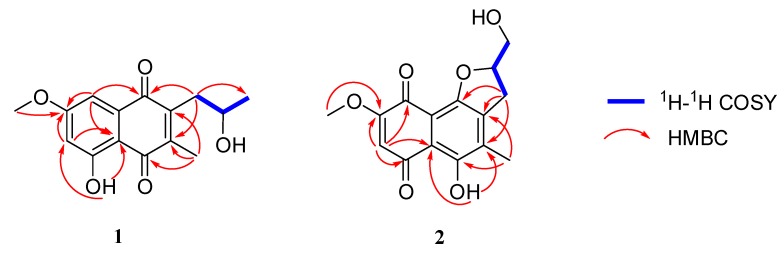
Key HMBC (red arrows) and COSY (blue bold lines) correlations of compounds **1** and **2.**

**Figure 3:**
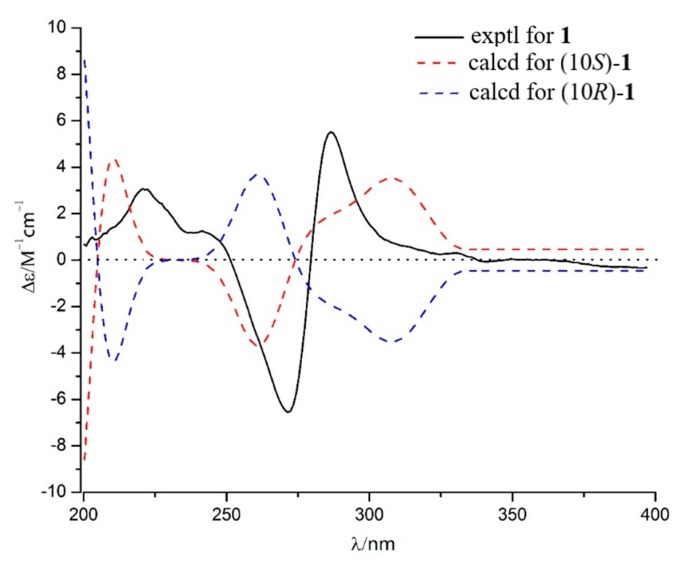
Calculated and experimental electronic circular dichroism (ECD) spectra of **1**.

Talanaphthoquinone A (**1**) was isolated as a yellow, amorphous powder. The high-resolution electrospray ionization mass spectrometry (HRESIMS) spectrum (see Supplementary Materials Figure S1) displayed a molecular ion peak at m/z 275.09223 [M − H]^–^ (calcd. for 275.09195), implying the molecular formula C_15_H_15_O_5_ (eight degrees of unsaturation). The ^1^H NMR spectrum (Table 1) showed signals for one phenolic hydroxyl proton *δ*_H_ 12.38 (s, HO-1), two meta-coupled aromatic protons (*δ*_H_ 7.17 (d, *J* = 2.5 Hz, H-4), *δ*_H_ 6.63 (d, *J* = 2.5 Hz, H-2)), two methyl groups (*δ*_H_ 2.21 (s, H_3_-12), *δ*_H_ 1.31 (d, *J* = 6.2 Hz, H_3_-11)), one methoxy (*δ*_H_ 3.90 (s, OCH_3_-13)), one oxygenated methine (*δ*_H_ 4.04 (dd, *J* = 11.9, 6.0 Hz, OCH-10)), and one methylene (*δ*_H_ 2.81 (s, H_a_-9), *δ*_H_ 2.80 (d, *J* = 1.7 Hz, H_b_-9)). The ^13^C NMR data (Table 1) revealed two carbonyls (*δ*_C_ 188.6, 185.2), eight sp^2^-hybridized olefinic or aromatic carbons (*δ*_C_ 165.9, 164.2, 145.7, 144.6, 133.7, 109.7, 107.8, 106.1), three methyl groups (*δ*_C_ 56.1, 12.9, 24.4), one methylene (*δ*_C_ 36.8), and one methine (*δ*_C_ 67.9). The planar structure of 1 was mainly established by HMBC and COSY correlations (Figure 2). The HMBC correlations from one aromatic proton H-2 (*δ*_H_ 6.63) to sp^2^ carbons C-3 (*δ*_C_ 165.9) and C-8a (*δ*_C_ 109.7), H-4 (*δ*_H_ 7.17) to C-2 (*δ*_C_ 106.1), C-3, C-5 (*δ*_C_ 185.2) and C-8a, led to the identification of the presence of a 1,3,4,5-tetrasubstituted benzene ring with a hydroxy, a methoxy, and two carbonyl groups attached at the C-1, C-3, C-4a, and C-8a, respectively. A methoxy group (*δ*_H_ 3.90, *δ*_C_ 56.1) was located at C-3, supported by its HMBC correlations with C-3. The HMBC correlations of the hydroxyl proton at *δ*_H_ 12.38 with C-2 and C-8a confirmed an angular 1, 4-napthaquinone structure having an intra-molecularly hydrogen-bonded hydroxyl group at C-1. The COSY correlations of H-10 with H_2_-9 and H_3_-11, combined with the chemical shift of C-10 (*δ*_C_ 67.9), showed the presence of one 2-hydroxypropyl group. The HMBC correlations from H-12 to C-6 (*δ*_C_ 144.6), C-7 (*δ*_C_ 145.7), and C-8 (*δ*_C_ 188.6), H-9 to C-5, C-6, and C-7 revealed the presence of another ring 1, 4-quinone, 2-hydroxypropyl group and CH_3_-12 were attached to the C-6 and C-7 of the napthaquinone ring, respectively. Mosher’s method was tried to identify the absolute configuration of C-10 [19]. Unfortunately, alpha-methoxy-alpha-trifluoromethylphenylacetic acid (MTPA) esters were not detected. As a result, the absolute configuration of C-10 was determined by comparing experimental and calculated electronic circular dichroism (ECD) spectra [20]. As shown in Figure 3, the calculated ECD curves of 10*S* was basically consistent with the CD curves obtained experimentally. Therefore, compound **1** was identified as shown in Figure 1.

Talanaphthoquinone B (**2**) was isolated as a red solid with ${\left[\alpha \right]}_{D}^{25}$: −16.8 (*c* 0.1, MeOH). The HRESIMS spectrum displayed a negative ion peak at m/z 307.08218 [M+H_2_O–H]^–^ (calcd. for C_15_H_15_O_7_, 307.08178), corresponding to the molecular formula C_15_H_14_O_6_, implying nine degrees of unsaturation. The UV and IR spectra of **2** were similar to those of talanaphthoquinone A, indicating that they were structurally related. Aside from the characteristic naphthoquinone NMR signals due to two quinone carbonyls (*δ*_C_ 190.1, 177.6) and eight aromatic/olefinic carbon resonances (*δ*_C_ 161.4, 157.3, 155.0, 139.0, 134.2, 110.3, 109.3, 109.1), ^1^H- and ^13^C-NMR spectrum (Table 1) showed the presence of signals due to two methyl groups (*δ*_H_ 3.88, *δ*_C_ 56.7, OCH_3_-13; *δ*_H_ 2.25, *δ*_C_ 13.3, CH_3_-12), two methylene groups (*δ*_H_ 3.20 (dd, *J* = 17.0, 9.3 Hz, 1H), *δ*_H_ 3.04 (dd, *J* = 16.6, 7.0 Hz, 1H), *δ*_C_ 29.9, CH_2_-9; *δ*_H_ 4.02 (d, *J* = 12.7 Hz, 1H), *δ*_H_ 3.76 (d, *J* = 8.7 Hz, 1H), *δ*_C_ 64.5, OCH_2_-11], an oxymethine group (*δ*_H_ 5.15 (d, *J* = 3.7 Hz), *δ*_C_ 86.2, OCH-10), one phenolic hydroxyl proton (*δ*_H_ 13.47 (s), HO-8), and one aromatic proton (*δ*_H_ 6.07 (s), H-2). The COSY correlations (Figure 2) of H-10 with H_2_-9 and H_2_-11 showed the presence of an independent spin coupling system -CH_2_-CH-CH_2_. The chemical shift of C-11 indicated one hydroxyl group was located on C-11. HMBC spectrum (Figure 2) showed that H-2 was correlated with C-1 (*δ*_C_ 190.1), C-3 (*δ*_C_ 161.4), C-4 (*δ*_C_ 177.6), and C-8a (*δ*_C_ 110.3), which could confirm that the only aromatic hydrogen was attached to the C-2 (*δ*_C_ 109.2). A methoxyl group was linked to olefinic carbon C-3, supported by the HMBC correlation of H-13 with C-3. The HMBC correlation of H-12 with C-6 (*δ*_C_ 139.0), C-7 (*δ*_C_ 134.2), and C-8 (*δ*_C_ 157.3) indicated that the methyl group was connected to the C-7. The HMBC correlation of H-9 with C-5 (*δ*_C_ 155.0), C-6, C-7, indicated that C-9 was attached to the C-6. Phenolic hydroxyl group was located at C-8, as indicated by the HMBC correlation with C-7 and C-8. The naphthoquinone structure unit explained seven of the eight degrees of unsaturation in **2**, implied by the molecular formula. The remaining one degree of unsaturation, together with downfield chemical shift of C-10 (*δ*_C_ 86.2), indicated that C-10 was attached to C-5 to fuse a furan ring. In the light of the chemical shift of C-11 (*δ*_C_ 64.5), the C-11 was a hydroxymethyl group. Therefore, the planar structure of compound **2** was elucidated as shown in Figure 1.

### 2.3. Inhibitory Effects on NO Production

All these isolates were tested for their inhibitory activities against lipopolysaccharide (LPS)-activated NO production in the murine macrophage cell line (RAW 264.7 cells). As shown in Table 2, all compounds could inhibit the production of NO with IC_50_ values ranging from 1.7 to 49.7 μM, while IC_50_ of indomethacin was 26.3 μM. It is suggested that the 1, 4-naphthoquinone is the active center of anti-inflammatory effect. Compound **2** has only one more hydroxyl group at position 11 than compound **3** in structure, but the inhibitory activity was apparently weaker, which indicates that the hydroxyl group at position 11 was not conducive to the anti-inflammatory activity. Their cytotoxic effects on RAW 264.7 cell lines was evaluated by the MTT assay. Six compounds (**2**–**6** and **8**) showed no cytotoxicity at a concentration of 50 μM. Six compounds (**1**, **7**, **9**, **10**, **11** and **12**) revealed some cytotoxicity with the selective index (SI = CC_50_/IC_50_) values from 2.1 to 29.6 (Table 2). The cytotoxicity data indicated that the third ring on the naphthoquinone can reduce the cytotoxicity to macrophage of mice. Compound **9** had lower cytotoxicity than **10**, which revealed that the hydroxyacetylation on the side chain can also reduce the cytotoxicity.

### 2.4. Inhibitory Effects on the Production of Inducible Nitric Oxide Synthase (iNOS), Cyclooxygenase-2 (COX-2), and Pro-Inflammatory Factors

**Figure 4:**
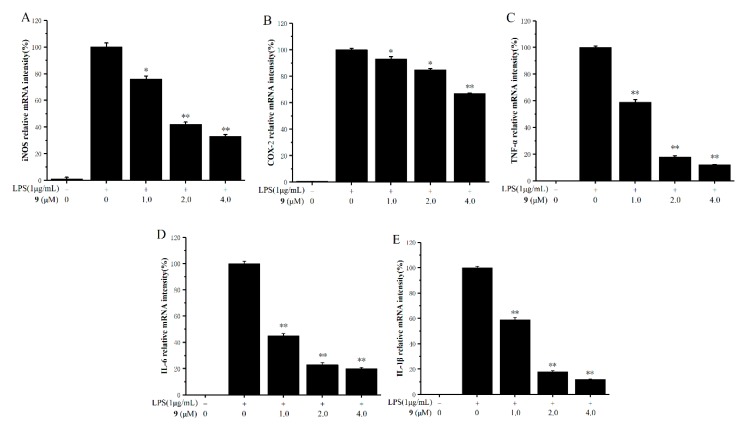
RAW 264.7 murine macrophage cells were pre-incubated for 12h, and then cells were pretreated with compound **9** at the indicated concentration for 1 h and incubated with LPS (1 μg/mL) for 12h (real-time PCR). The effect of compound 9 on the mRNA expressions of inducible nitric oxide synthase (iNOS) (**A**), cyclooxygenase-2 (COX-2)(**B**), tumor necrosis factor (TNF)-α (**C**), interleukin (IL)-6 (**D**), and IL-1β (**E**) were detected by real-time PCR. The data represent the mean values ± SD of three experiments, * *p* < 0.05, ** *p* < 0.01 compared to LPS-treated group.

Based on the results of inhibitory effects on NO production, compound **9** was found to be a significant inhibitor of NO with low cytotoxicity (SI = 29.6). We have further investigated the effects of CC_50_/IC_50_ on mRNA expressions of inducible nitric oxide synthase (iNOS), cyclooxygenase-2 (COX-2), and the pro-inflammatory cytokines tumor necrosis factor (TNF)-α, interleukin (IL)-1β, and IL-6 in LPS-stimulated RAW 264.7 cells using real-time polymerase chain reaction (RT-PCR) analysis. As shown in Figure 4A–E, compound **9** apparently reduced the mRNA expressions of iNOS, COX-2, TNF-α, IL-1β, and IL-6 in a dose-dependent manner. Especially at the concentration of 2.0 μM, compound **9** inhibited the mRNA expressions of TNF-α, IL-1β, and IL-6 by about 80% compared with the control groups.

## 3. Materials and Methods

### 3.1. General Experimental Procedures

The 1D and 2D NMR data were recorded on Bruker Avance 400 and 500 spectrometer (Bruker BioSpin Corporation, Billerica, MA, USA), in which all chemical shifts (*δ*) are given in ppm with reference to tetramethylsilane (TMS), and coupling constants (*J*) are given in Hz. EIMS data were measured on a dual stage quadrupole electron impact (DSQ EI)-mass spectrometer (Thermo, Shanghai, China) and the high-resolution electrospray ionization mass spectrometry (HRESIMS) were determined with a quadrupole time of flight (Q-TOF) high-resolution mass spectrometer (Waters Corporation, Synapt G2-Si, Milford, MA, USA). UV data were measured on a UV-240 spectrophotometer (Shimadzu, Beijing, China). IR data were recorded on a Nicolet 5DX-FTIR (Thermo Fisher Scientific, Inc., Hudson, NH), in KBr discs. Melting points were measured on an X-4 micromelting-point apparatus (Cany Precision Instruments Co., Ltd., Shanghai, China, uncorrected). Column chromatography (CC) was carried out on silica gel (200–300 mesh, Qingdao Marine Chemical Factory, Qingdao, China) and Sephadex LH-20 (Amersham Pharmacia, Piscataway, NJ, USA). GF-254 precoated silica gel plates (Qingdao Huang Hai Chemical Group Co., Qingdao, China) were used for thin layer chromatography. Semipreparative HPLC separation was performed on a Hitachi Primaide 1430 HPLC system (HITACHI, Tokyo, Japan) using a C_18_ column (Phenomenex, Torrance, CA, USA; 250 × 10 mm, 5 μm) with the flow rate of 2.0 mL/min.

### 3.2. Fungal Material

The fungal strain used in this study was isolated from the fresh fruit of *Kandelia obovata*, which was collected from Shankou National Mangrove Nature Reserve in the South China Sea in September 2013. It was separated and identified by Senhua Chen using the standard protocol [21]. The sequence data obtained from the fungal strain were deposited at Gen Bank with accession no. MK368459. A BLAST search result revealed that the sequence was the most similar (100%) to the sequence of *Talaromyces amestolkiae* (KT445914.1). A voucher strain was deposited in School of Chemistry, Sun Yat-Sen University, Guangzhou, China.

### 3.3. Extraction and Isolation

The fungus *Talaromyces* sp. SK-S009 was fermented on autoclaved rice solid-substrate medium in 80 500-mL Erlenmeyer flasks (each containing 50 g rice and 50 mL water with 3‰ of saline) for 28 days at 25 °C. Following incubation, the mycelia and solid rice medium were extracted with EtOAc. The organic solvent was filtered and concentrated under reduced pressure to yield 40 g organic extract. The extract was subjected to silica gel CC using gradient elution with petroleum ether-EtOAc from 90:10 to 0:100 (v/v) to give 14 fractions (fractions1–14). Fraction 3 (600 mg) was applied to Sephadex LH-20 CC and eluted with CHCl_3_/MeOH (1:1) to obtain seven subfractions (fractions 3.1–3.7). Fraction 3.4 (100 mg) was further purified by RP-HPLC (70% MeOH in H_2_O) to afford **5** (12.1 mg, *t*_R_ = 10.0 min) and **1** (3.6 mg, *t*_R_ = 15.6 min). Fraction 3.7 (41 mg) was further purified by RP-HPLC (65% MeOH in H_2_O) to afford **7** (5.8 mg, *t*_R_ = 22.0 min). Fraction 5 (560 mg) was further purified by RP-HPLC (70% MeOH in H_2_O) to afford **12** (10.8 mg, *t*_R_ = 15.2 min), **3** (15.4 mg, *t*_R_ = 18.6 min), and **11** (8.6 mg, *t*_R_ = 26.1 min). Fraction 7 (503 mg) was further purified by silica gel CC using 80% EtOAc-light petroleum to afford seven subfractions (fractions 7.1–5.7). Fraction 7.3 (90 mg) was further purified by RP-HPLC (60% MeOH in H_2_O) to afford 4 (20 mg, *t*_R_ =14.5 min). Fraction 8 (870 mg) was applied to Sephadex LH-20 CC, eluted with CHCl_3_/MeOH (1:1), to obtain 10 subfractions (fractions 8.1–8.10). Fraction 8.5 (25 mg) was further purified by Sephadex LH-20 CC, eluted with CHCl_3_/MeOH (1:1), to afford **2** (3.3 mg). Fraction 8.7 (40 mg) was further purified by Sephadex LH-20 CC, eluted with CHCl_3_/MeOH (1:1), to afford **6** (13.3 mg). Fraction 9 (680 mg) was applied to silica gel CC using 60% EtOAc-petroleum ether to afford seven subfractions (fractions 9.1–9.7). Fraction 10.3 was further purified by RP-HPLC (50% MeOH in H_2_O) to afford **8** (7.3 mg, *t*_R_ = 12.5 min), **9** (9.1 mg, *t*_R_ = 18.1 min), and **10** (20 mg, *t*_R_ = 20.4 min).

#### 3.3.1. Talanaphthoquinone A (**1**)

Yellow powder ${\left[\alpha \right]}_{D}^{25}$: −17.0 (*c* 0.1, MeOH); IR (KBr) ν_max_: 3375, 1655, 1633, 1602, 1576, 1379, 1302, 1207, 1167, 1113, 954, 817, 773 cm^−1^. UV (MeOH) *λ*_max_ (log *ε*): 268 (2.34), 219 (3.63) nm. The ^1^H and ^13^C NMR spectroscopic data, see Table 1; HRESIMS (m/z 275.09223 [M − H]^−^, calcd. for C_15_H_15_O_5_, 275.09195).

#### 3.3.2. Talanaphthoquinone B (**2**)

Red solid ${\left[\alpha \right]}_{D}^{25}$: −16.8 (c 0.1, MeOH); m.p. 157.7–159.3 °C; IR (KBr) ν_max_: 3422, 2981, 1672, 1636, 1587, 1473, 1452, 1252, 1221, 1165, 1091, 1031, 962, 930, 886, 863, 816, 790 cm^-1^. UV (MeOH) *λ*_max_ (log *ε*): 300 (2.45), 225 (3.89) nm. The ^1^H and ^13^C NMR spectroscopic data, see Table 1; HRESIMS (m/z 307.08218 [M+H_2_O-H]^−^, calcd. for C_15_H_15_O_7_, 307.08178).

### 3.4. Measurement of NO Production and Cell Viability

The nitrite/nitrate oxidized from NO was detected by the Griess reagent as previously reported with slight modifications [22,23]. The murine macrophage cell line (RAW 264.7 cells) were seeded in 96-well plates at a density of 1 × 10^5^ cells/well and preincubated for 12 h. The tested compounds were dissolved in dimethylsulfoxide (DMSO) and diluted with Dulbecco’s Modified Eagle Medium (DMEM) medium to a final concentration (final DMSO concentration <0.1% in all assays). Indomethacin was used as the positive control. Cells were stimulated with 1µg/mL of lipopolysaccharide (LPS, Sigma, St. Louis, MO, USA) with or without tested compounds for 24 h. Then culture supernatant was mixed with an equal volume of Griess reagent I and II (Beyotime, Shanghai, China), and measured at 540 nm using a microplate reader (Bio-Rad Laboratories, Inc., Kyoto, Japan). Cell viability was evaluated using MTT assay [24].

### 3.5. Real-Time PCR

RAW264.7 cells were placed in a 6-well plate at a density of 1 × 10^6^ cells/well and incubated for 12 h. Cultured cells were pretreated with compound **9** (1, 2, 4 μM/L) for 1 h and incubated with LPS (1 μg/mL) for 12 h. RNA was extracted with Trizol Reagent (Takara Bio Inc., Otsu, Japan) according to the manufacturer’s instructions, and cDNA was reverse transcribed from total RNA using a Superscript III system (Takara Bio Inc., Otsu, Japan). PCR amplification was carried out using PikoReal™ (Thermo Fisher Scientific, MA, US) and specific primers. The primer sequences (Wcgene Biotech, Shanghai, China) are shown in Table 3. The optimal conditions for PCR amplification of the cDNA were established by following the manufacturer’s instructions. Relative gene expression was calculated using the comparative Ct method (2^−ΔΔCt^) with glyceraldehyde-3-phosphate dehydrogenase (GAPDH) as an internal control. All experiments were performed in triplicate (*n* = 3).

## 4. Conclusions

The 1, 4-naphthoquinone derivatives were reported to show potential anti-inflammatory effect, including reducing 12-O-tetradecanoylphorbol-13-acetate (TPA)-induced acute inflammation in mouse ear [25], suppressing the production of TNF-α induced by LPS in mouse macrophages [25]. Suppressing TNF-α production in serum in vivo mouse model of LPS evoked acute inflammation [25], reducing cotton pellet- and carrageenin-induced paw edema in rat [26], and so on.

In this study, we isolated two new 1, 4-naphthoquinone derivatives, talanaphthoquinone A, B (**1**, **2**), along with 10 known analogues (**3**–**12**) from mangrove-derived endophytic fungus *Talaromyces* sp. SK-S009. All the compounds strongly inhibited LPS-induced NO production in RAW264.7 cell line in a dose-dependent manner. The NO production inhibition activities of all the compounds except for compound **2** were more potent than the positive control, indomethacin. Six compounds (**1**, **7**, **9**, **10**, **11**, and **12**) showed relatively less cytotoxicity to RAW264.7 cells with SI values varied from 2.1 to 29.6. A primary analysis of the structure−activity relationships was discussed. Furthermore, compound **9** reduced the mRNA levels of iNOS, COX-2, IL-1β, IL-6, and TNF-α. The results of this study demonstrated that these 1, 4-naphthoquinone derivatives can inhibit LPS-induced inflammation. This is the first report that compound **9** possesses anti-inflammatory activity.

## Figures and Tables

**Table 1:** The ^1^H and ^13^C NMR data (CDCl_3_, 500/125 MHz) of compounds **1** and **2**.

Position	1		2	
	*δ*_C_	*δ*_H_ (*J* in Hz)	*δ*_C_	*δ*_H_ (*J* in Hz)
1	164.2, C	-	190.1, C	-
2	106.1, C	6.63, d (2.5)	109.2, CH	6.07, s
3	165.9, C	-	161.4, C	-
4	107.8, C	7.17, d (2.5)	177.6, C	-
4a	133.7, C	-	109.1, C	-
5	185.2, C	-	155.0, C	-
6	144.6, C	-	139.0, C	-
7	145.7, C	-	134.2, C	-
8	188.6, C	-	157.3, C	-
8a	109.7, C	-	110.3, C	-
9	36.8, CH_2_	2.81, s 2.80, d (1.7)	29.9, CH_2_	3.04, dd (7.1,16.6); 3.20, dd (9.4,17.0)
10	67.9, CH	4.04, dd (11.9, 6.0)	86.2, CH	5.15, m
11	24.4, CH_3_	1.31, d (6.2)	64.5, CH_2_	3.76, d (8.6); 4.02, d (12.7)
12	12.9, CH_3_	2.21, s	13.3, CH_3_	2.25, s
13	56.1, CH_3_	3.9, s	56.7, CH_3_	3.88, s
1-OH	-	12.38, s	-	13.47, s

**Table 2:** Inhibitory activity of all compounds **1**−**12** against lipopolysaccharide (LPS)-induced NO production in the murine macrophage cell line (RAW 264.7 cells).

Compounds	IC_50_ (μM)	CC_50_ (μM) ^a^	SI ^b^
1	3.9 ± 0.5	30.7 ± 0.5	7.9
2	49.7 ± 1.5	-	
3	16.0 ± 0.2	-	
4	22.6 ± 0.5	-	
5	11.2 ± 0.3	-	
6	5.2 ± 0.1	-	
7	14.4 ± 0.6	51.4 ± 1.5	3.6
8	7.7 ± 0.3	-	
9	1.7 ± 0.2	50.3 ± 1.5	29.6
10	7.5 ± 0.2	15.8 ± 0.4	2.1
11	15.5 ± 0.6	59.2 ± 1.5	3.8
12	5.6 ± 0.3	48.4 ± 1.3	8.6
Indomethacin ^c^	26.3 ± 0.6		

^a^ Values are taken as the means ± standard deviation, *n* = 3; ^b^ SI, selectivity index, calculated by CC_50_ /IC_50_; ^c^ Positive control; - No cytotoxicity was observed at a concentration of 50 μM.

**Table 3:** Primer sequences in this experiment.

Primer		Primer Sequence (5′ to 3′)
iNOS	Forward	GTCTTTGACGCTCGGAACTGTAG
	Reversed	TGAAGTCATGTTTGCCGTCACT
COX-2	Forward	GATGACTGCCCAACTCCC
	Reversed	AACCCAGGTCCTCGCTTA
TNF-α	Forward	TGGCTGCTGAAAAGACACATGT
	Reversed	CCACCAGACGTTCTGCTGTCTAG
IL-1β	Forward	AGTTGACGGACCCCAAAAG
	Reversed	AGCTGGATGCTCTCATCAGG
IL-6	Forward	TTCCATCCAGTTGCCTTCTTG
	Reversed	GGGAGTGGTATCCTCTGTGAAGTC
GADPH	Forward	TGTGTCCGTCGTGGATCTGA
	Reversed	TTGCTGTTGAAGTCGCAGGAG

## Supplementary Materials

The following are available online, Figures S1–S12: HRESIMS spectrum, 1D- and 2D-NMR spectra of 1 and 2.
