# Supplementary material for: Naphthoquinone Derivatives with Anti-Inflammatory Activity from Mangrove-Derived Endophytic Fungus Talaromyces sp. SK-S009
Source: Molecules. 2020 Jan 29;25(3):576. doi: 10.3390/molecules25030576 (PMC7037671; doi:10.3390/molecules25030576)
Supplement: Supplementary file 1 [file molecules-25-00576-s001.pdf]

SUPPORTING INFORMATION

Naphthoquinone Derivatives with Anti-inflammatory Activity from Mangrove Derived Endophytic Fungus *Talaromyces* sp. SK-S009

Hongju Liu [a,*], Chong Yan [a], Changqun Li [a], Tingting You [a] and Zhigang She [b,*]

[a] School of Pharmacy, Guangdong Medical University, Dongguan 523808, China;

[b] School of Chemistry, Sun Yat-Sen University, Guangzhou 510275, China;

*Corresponding authors: Hongju Liu (Email: liuhj8@mail2.sysu.edu.cn); Zhigang She (Email: cesshzhg@mail.sysu.edu.cn; Tel/Fax: +86-20-84113356)

Table of contents:

Figure S1. HRESIMS spectrum of compound **1**

Figure S2. 1H NMR (500 MHz, CDCl3) spectrum of compound **1**

Figure S3. ^{13}C NMR (125MHz, $CDCl_3$) spectrum of compound **1**

Figure S4. ^{1}H–^{1}H COSY (500 MHz) spectrum of Compound **1** in $CDCl_3$

Figure S5. HSQC (500 MHz) spectrum of Compound **1** in $CDCl_3$

Figure S6. HMBC (500 MHz) spectrum of Compound **1** in $CDCl_3$

Figure S7. HREIMS spectrum of compound **2**

Figure S8. ^{1}H NMR (500 MHz, $CDCl_3$) spectrum of compound **2**

Figure S9. ^{13}C NMR (125MHz, $CDCl_3$) spectrum of **2**

Figure S10. ^{1}H–^{1}H COSY (500 MHz) spectrum of Compound **2** in $CDCl_3$

Figure S11. HSQC (500 MHz) spectrum of Compound **2** in $CDCl_3$

Figure S12. HMBC (500 MHz) spectrum of Compound **2** in $CDCl_3$

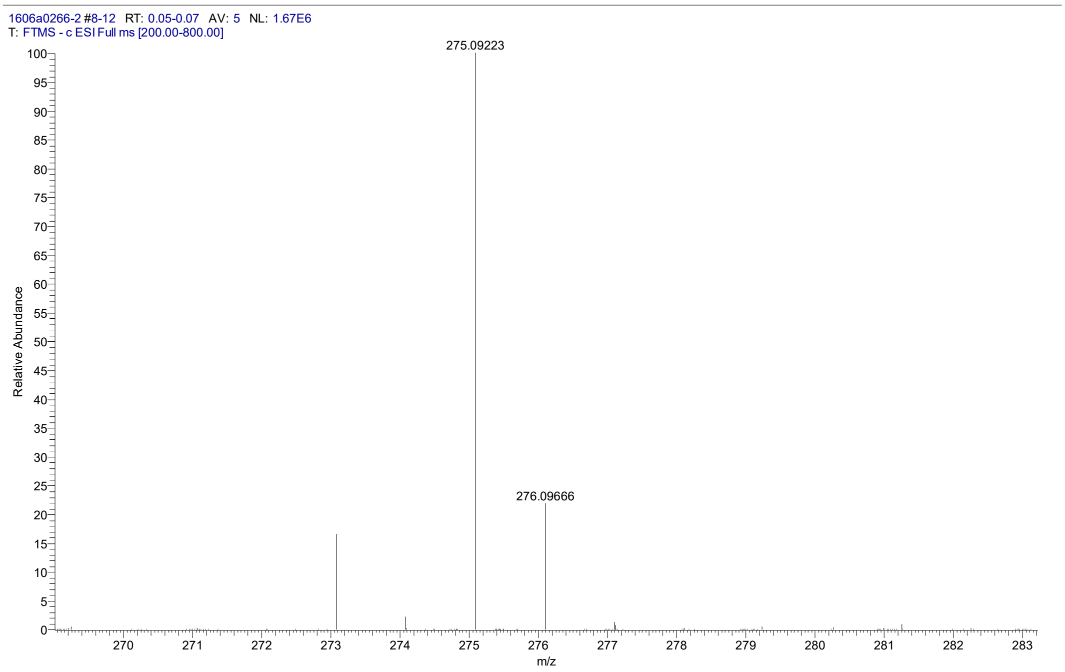

Figure S1. HRESIMS spectrum of compound **1**.

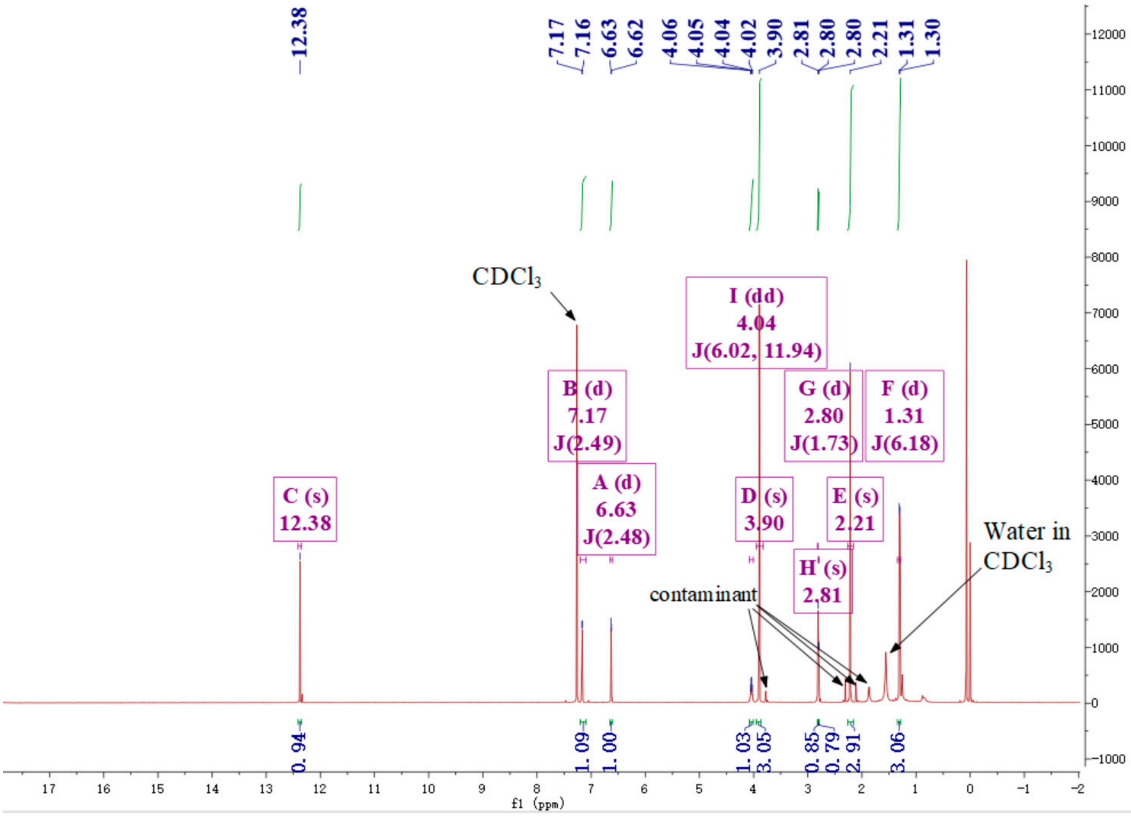

Figure S2. ^{1}H NMR (500 MHz, $CDCl_3$) spectrum of compound **1**.

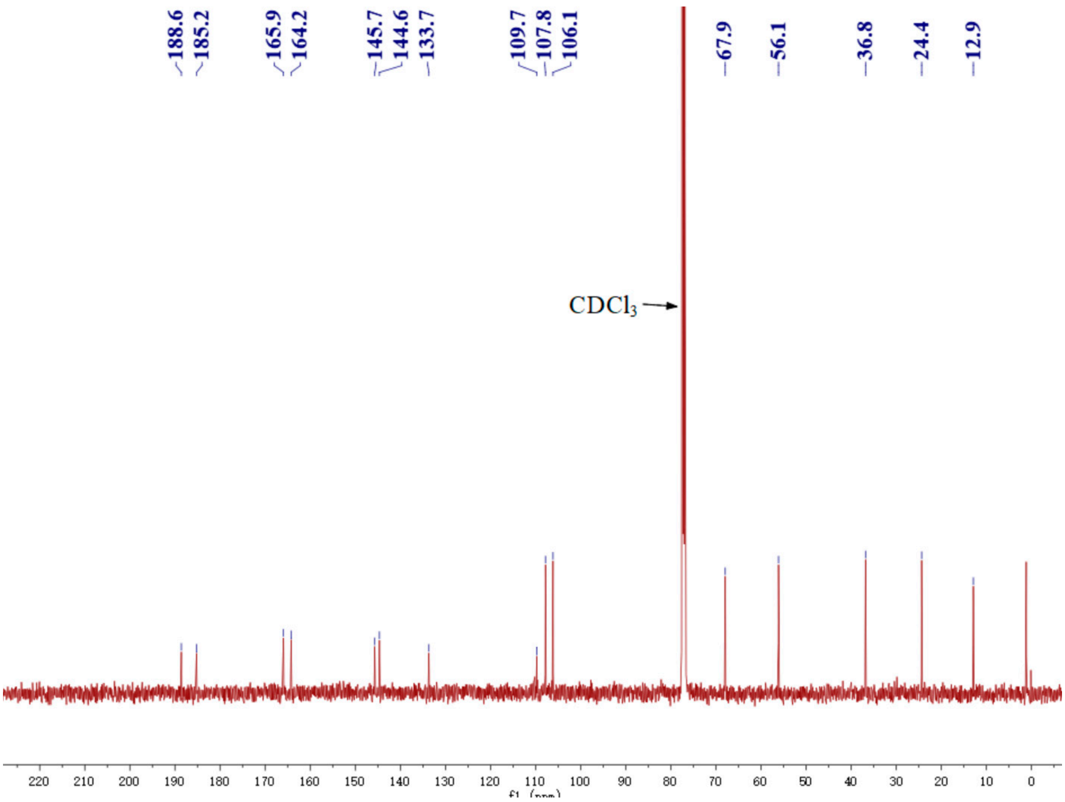

Figure S3. ^{13}C NMR (125 MHz, $CDCl_3$) spectrum of compound **1**.

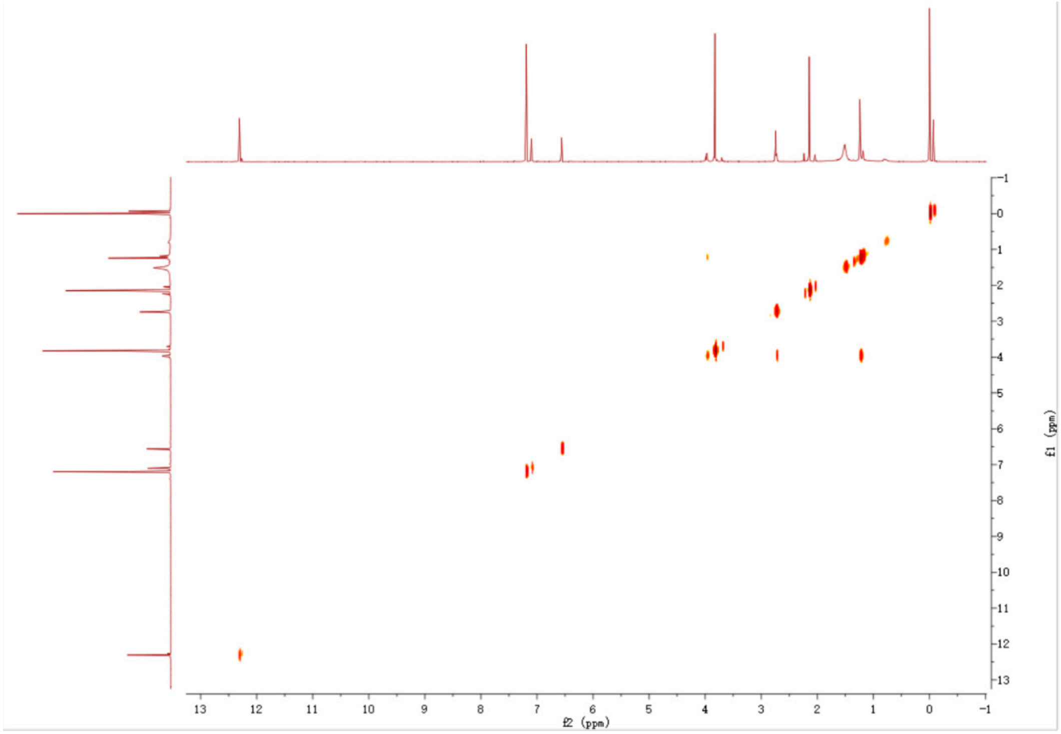

Figure S4. $^{1}H–^{1}H$ COSY spectrum of Compound **1** in $CDCl_3$.

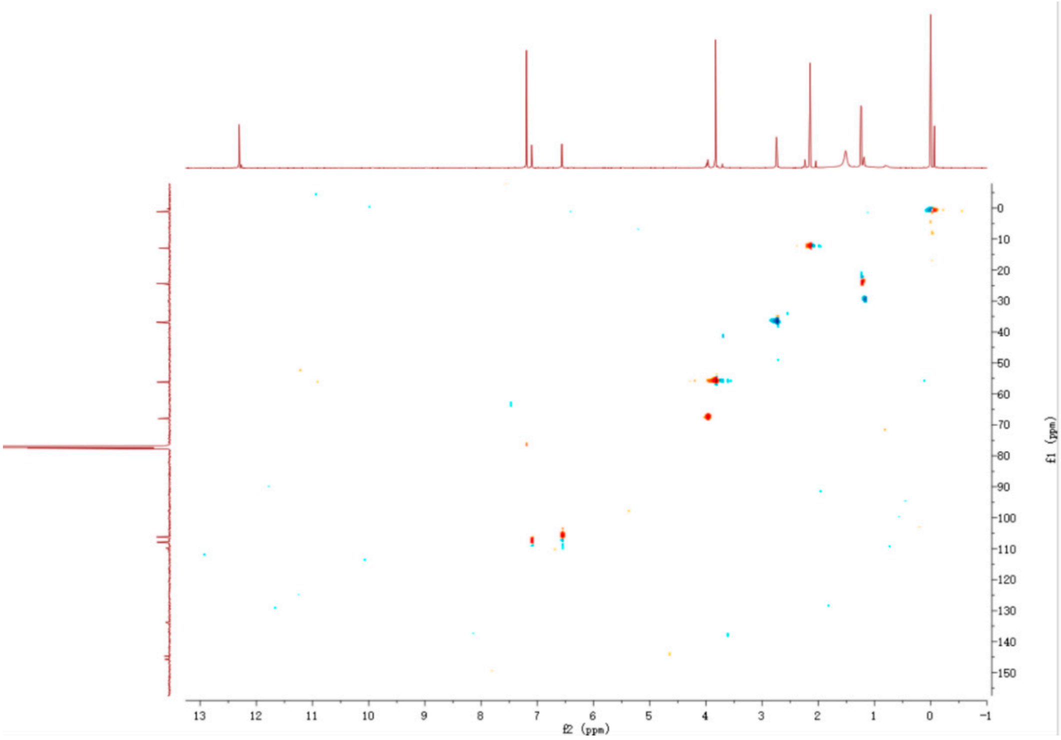

Figure S5. HSQC spectrum of Compound **1** in $CDCl_3$.

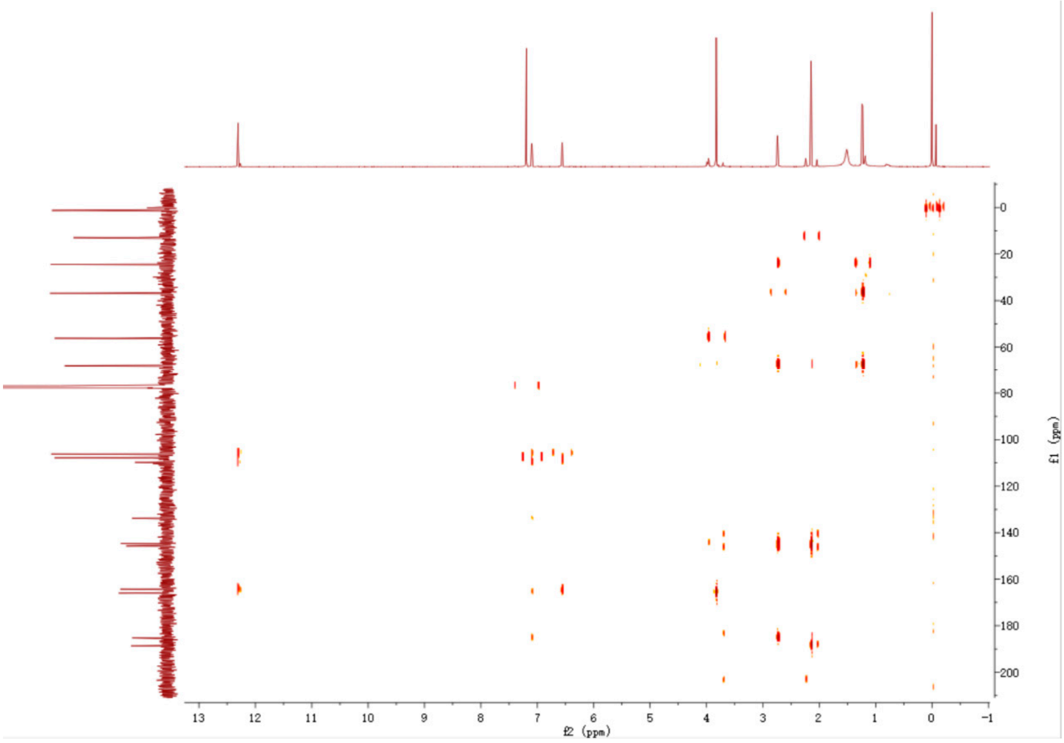

Figure S6. HMBC spectrum of Compound **1** in $CDCl_3$.

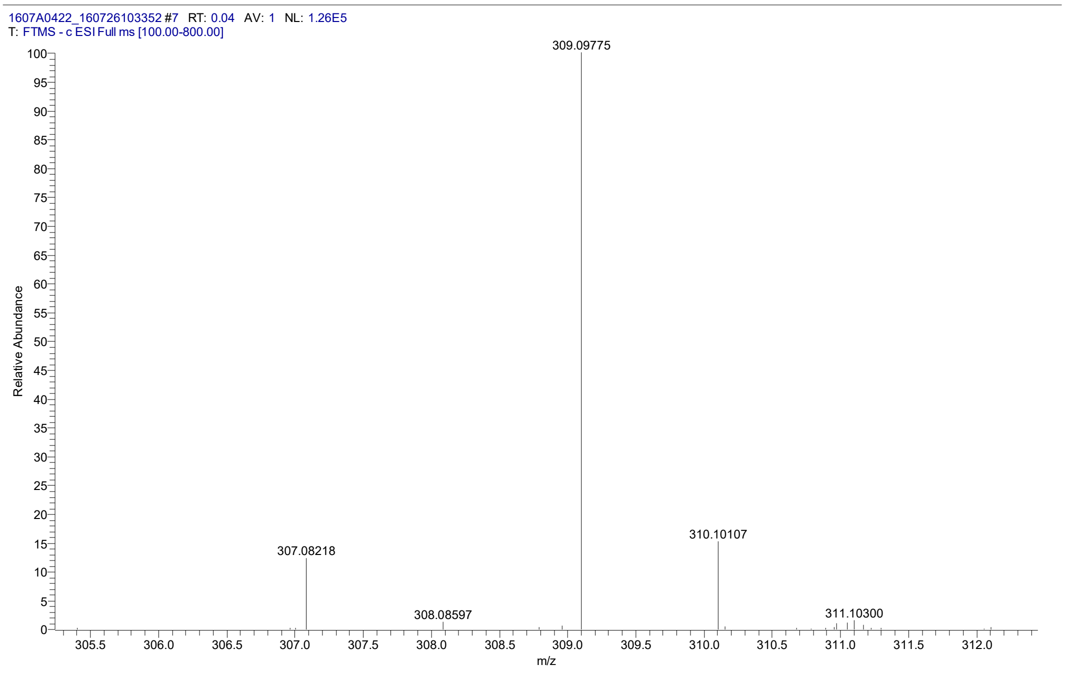

Figure S7. HRESIMS spectrum of Compound **2**.

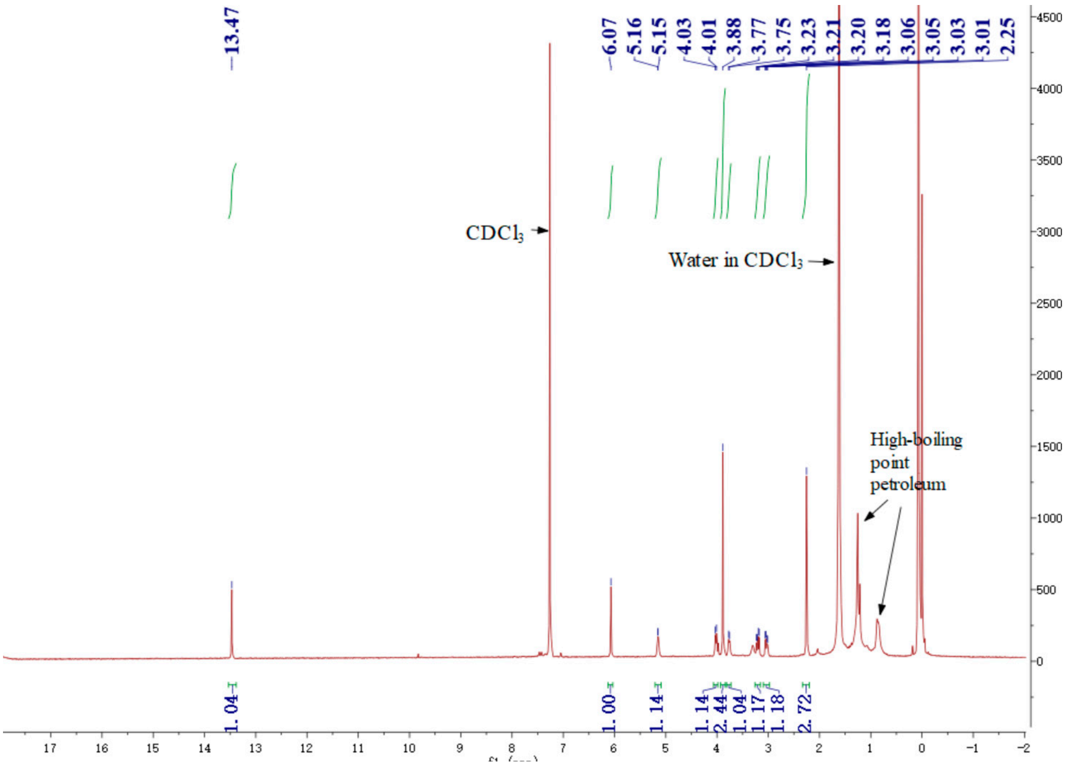

Figure S8. ^{1}H NMR (500 MHz, $CDCl_3$) spectrum of Compound **2**.

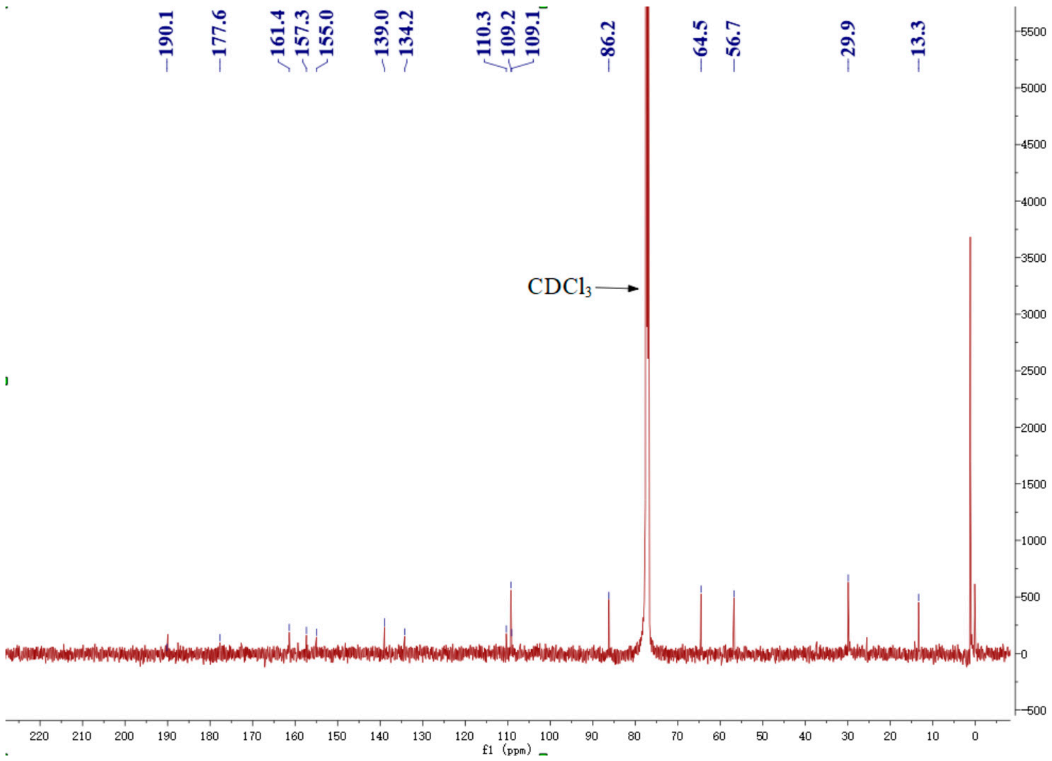

Figure S9. ^{13}C NMR (125 MHz, $CDCl_3$) spectrum of Compound 2.

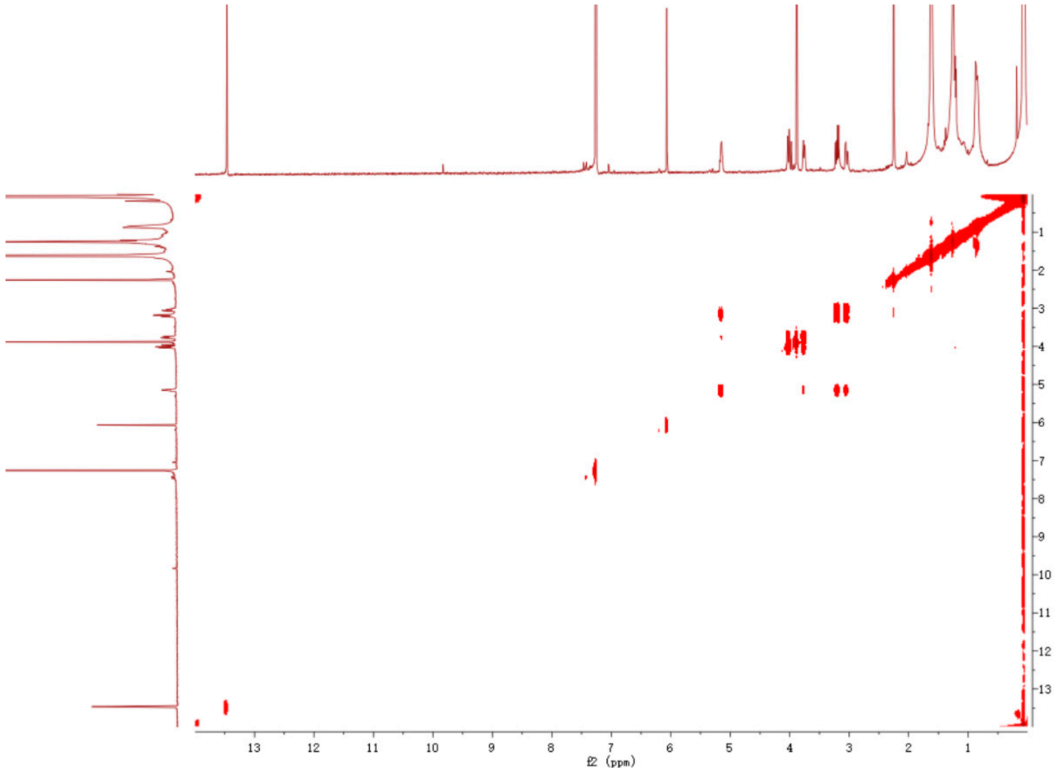

Figure S10. ^{1}H–^{1}H COSY spectrum of Compound **2** in $CDCl_3$.

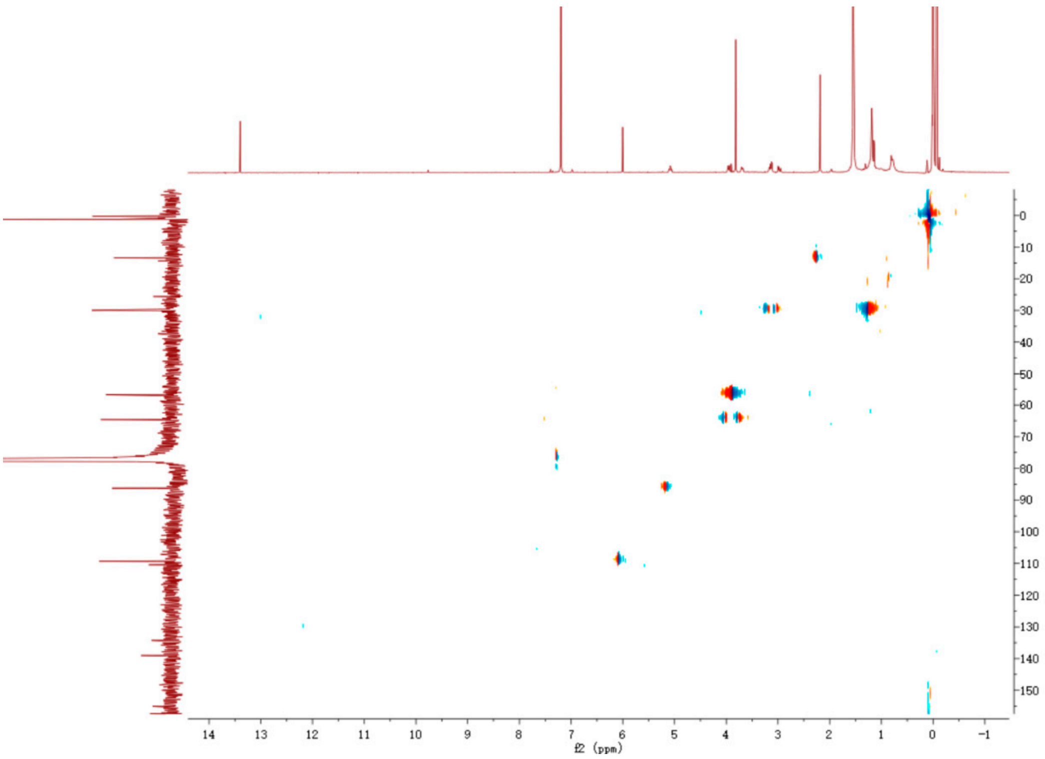

Figure S11. HSQC spectrum of Compound **2** in $CDCl_3$.

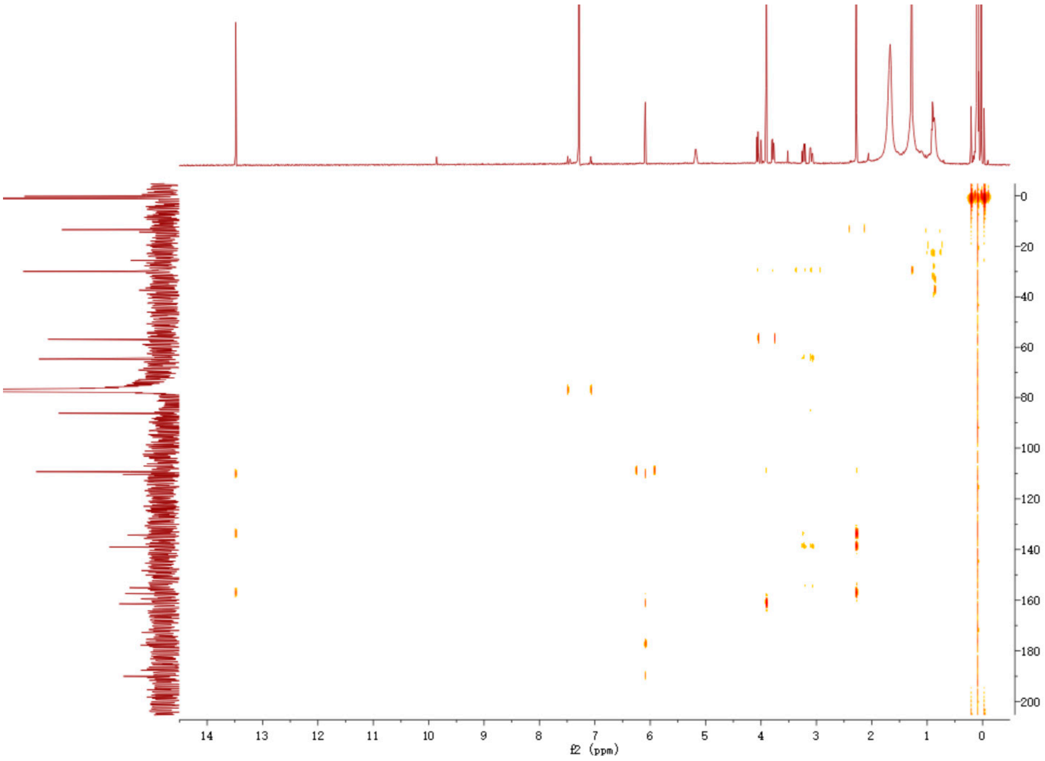

Figure S12. HMBC spectrum of Compound **2** in $CDCl_3$.
